# Hierarchical Novelty-Familiarity Representation in the Visual System by Modular Predictive Coding

**DOI:** 10.1371/journal.pone.0144636

**Published:** 2015-12-15

**Authors:** Boris Vladimirskiy, Robert Urbanczik, Walter Senn

**Affiliations:** Department of Physiology, University of Bern, Bühlplatz 5, 3012 Bern, Switzerland; SUNY Downstate MC, UNITED STATES

## Abstract

Predictive coding has been previously introduced as a hierarchical coding framework for the visual system. At each level, activity predicted by the higher level is dynamically subtracted from the input, while the difference in activity continuously propagates further. Here we introduce modular predictive coding as a feedforward hierarchy of prediction modules without back-projections from higher to lower levels. Within each level, recurrent dynamics optimally segregates the input into novelty and familiarity components. Although the anatomical feedforward connectivity passes through the novelty-representing neurons, it is nevertheless the familiarity information which is propagated to higher levels. This modularity results in a twofold advantage compared to the original predictive coding scheme: the familiarity-novelty representation forms quickly, and at each level the full representational power is exploited for an optimized readout. As we show, natural images are successfully compressed and can be reconstructed by the familiarity neurons at each level. Missing information on different spatial scales is identified by novelty neurons and complements the familiarity representation. Furthermore, by virtue of the recurrent connectivity within each level, non-classical receptive field properties still emerge. Hence, modular predictive coding is a biologically realistic metaphor for the visual system that dynamically extracts novelty at various scales while propagating the familiarity information.

## Introduction

A major challenge in understanding the human connectome is to unravel the intimate relationship between anatomical and effective (functional) connectivity [1–4]. It has been recognized that effective connectivity in terms of correlated activity does not necessarily require direct anatomical projections [5]. On the other hand, anatomical connectivity in the form of white matter tracts has been found to imply effective connectivity [6]. However, as we point out here, this does not hold for anatomical projections in general. Even if excitatory connections instantaneously drive the postsynaptic activity, in a recurrent network the activity on the time scale of network dynamics may be causally unrelated to the instantaneous drive.

**Fig 1:**
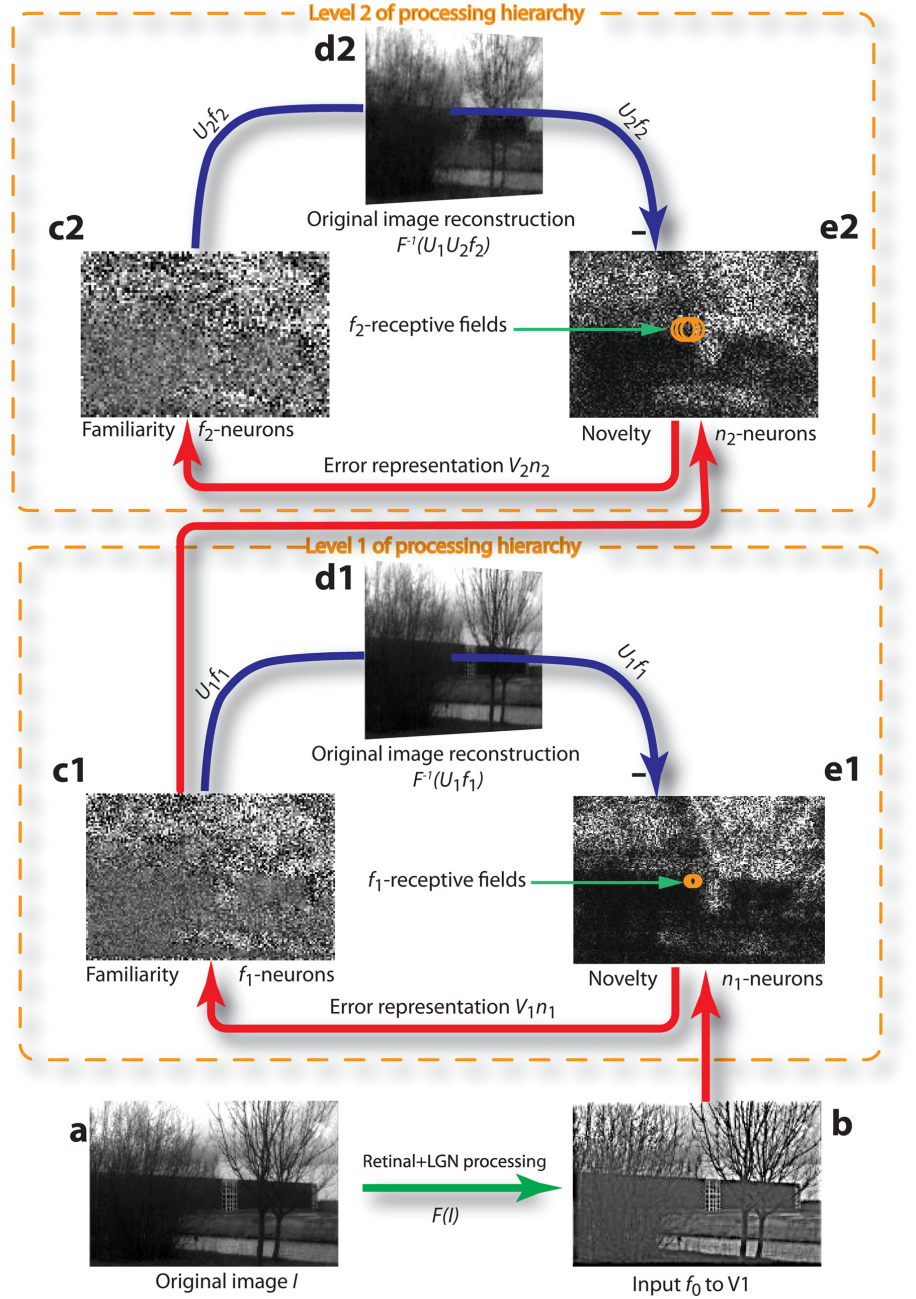
Modular predictive coding and image reconstruction after learning. **a** Example of a novel image *I* presented to the network after learning on 1000 other images in a total of 5000 randomly sampled presentations. **b** Input *f*_0_ to the first level after retinal and LGN processing. **c1** Activity of level-1 familiarity neurons *f*_1_ receiving 1-to-1 input from *n*_1_-neurons through constrained connection matrix *V*_1_ ($\approx U_{1}^{T}$). **d1** Reconstruction of the preprocessed image based on the steady-state activity of the level-1 familiarity neurons, *f*_0_ ≈ *U*_1_ *f*_1_. **e1** Activity of level-1 novelty neurons *n*_1_ receiving 1-to-1 input from the LGN and localized input from *f*_1_-neurons through the constrained connection matrix *U*_1_. Orange circles represent RFs of three neighboring *f*_1_-neurons. **c2, d2, e2** The corresponding quantities for level 2. The number of *f*_2_-neurons is 50% of the number of *f*_1_-neurons and 25% of the number of *n*_1_-neurons (= number of pixels in the visual input).

**Fig 2:**
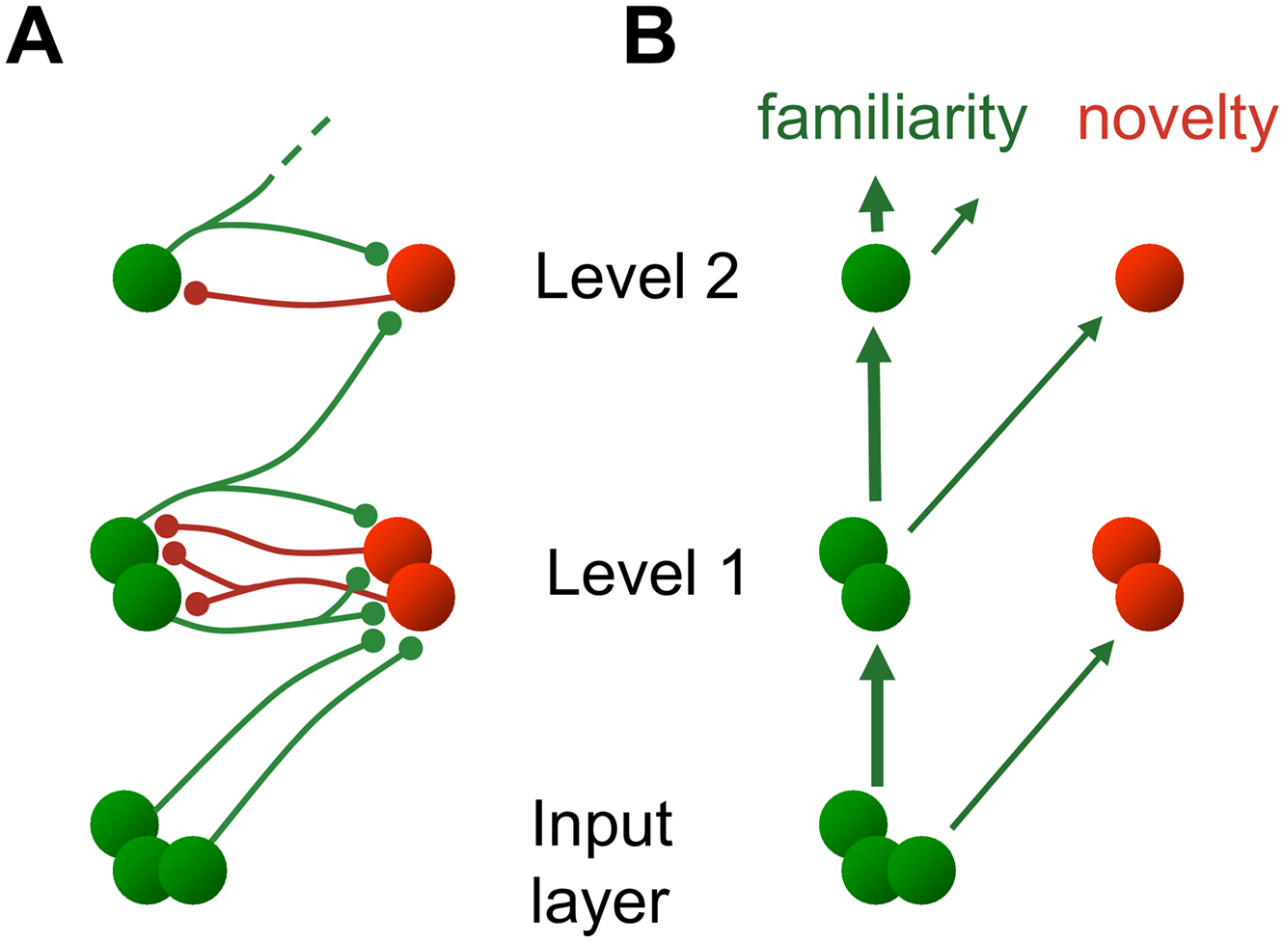
Anatomical versus effective connectivity. **A** Schematic anatomical connectivity pattern in the early ventral visual cortex shows recurrent synaptic connections within each level (Eq (4)). Lower-level ‘familiarity’ neurons (green) project to ‘novelty’ neurons (red) at the next higher level. **B** Effective connectivity expressing causal relationships results in a purely feedforward network (Eq (5)). At each level, familiarity and novelty information is extracted from the familiarity representation at the previous level.

Our model of stimulus representation in the visual system provides an example of how effective and anatomical connectivities may differ. The visual system has been described as a hierarchy of predictive coding schemes, where activity in a lower level is ‘predicted’ by activity in the next level of the hierarchy [7–9]. Because the ‘prediction’ in the higher level is effectively subtracted from the lower-level activity, it was argued that in this lower level only the error signal remains, and, as a consequence, only novelty information is passed to higher cortical levels [7, 10]. However, this conclusion appears to rely on the short-term network dynamics only. Instead, we propose that once the recurrent network has equilibrated, it is in fact the familiarity information, not the novelty, that is projected forward to higher cortical areas (see also [11]). We show that at each level of the hierarchy the recurrent dynamics divides the input signal into orthogonal familiarity and novelty components. Both of these components do effectively only depend on the lower-level familiarity component, but this functional dependence is not reflected in the direct anatomical connectivity (cf. Figs 1 and 2). This is where our approach differs crucially from the previous hierarchical predictive-coding work [7, 11], in which explicit feedback connections from higher cortical areas played an essential role in the network functionality.

To link anatomical and effective connectivity one may start with either of them. Classically, data on anatomical connectivity is first collected and then interpreted in functional terms. From a theoretical point of view, however, it is natural to first consider a possible functional purpose, and then seek its neuronal implementation. Generative models represent a general framework for operating in this opposite direction [12, 13]. The basic idea is to specify a model describing how sensory stimuli can be reconstructed (‘generated’) from a lower-dimensional neuronal activity pattern. The function assigned to the visual system in this setting is to represent visual stimuli in a compressed form such that the original stimuli can be reconstructed as closely as possible (see also [14, 15]). This approach is also adopted in predictive coding [7] and can itself be deduced from a unifying Bayesian optimization principle [16]. Given a generative model for reconstructing an image *I* from neuronal firing rates *f*, *I* ≈ Φ(*f*), one may ask whether the firing rates in turn could be explained by some neuronal processing triggered by the image. This amounts to inverting the generative model and obtaining the firing rates from the image, *f* ≈ Φ^−1^(*I*). The task is then to find a generative function Φ such that its inverse Φ^−1^ can be implemented in a neuronal circuitry. We show that the requirement of neuronal implementability of the inverse generative function strongly constrains the neuronal transfer function, essentially only allowing threshold-linear neurons. Further restrictions on the generative model arise from the fact that the receptive fields of neurons have limited size; thus, additional neuronal layers are required to extract more global features from visual stimuli.

Here we suggest that stimulus representation and recognition in the visual system is based on a modular hierarchy of predictive coding schemes with an effectively feedforward character. Recurrent connections are restricted to each individual level to separate novelty from familiarity information; they do not feed back to preceding levels as originally suggested [7]. We show that a quadratic regularity constraint can make this modular architecture functionally very similar to the fully recurrent architecture while keeping the advantages of a level-specific optimal encoding and the fast relaxation time characteristic of visual perception [17–19]. Furthermore, non-classical receptive field (RF) properties—as observed in the original predictive coding scheme [7, 20]—emerge from the within-level lateral connections, despite the absence of the top-down projections and despite linear neuronal dynamics only. This becomes possible because the RFs are limited to small overlapping areas on which the neurons develop specific interactions.

Finally, we suggest that, instead of being essential to the representation of natural visual stimuli by means of predictive coding, top-down connections are engaged in attention, memory recall and in top-down-gated learning, without substantially affecting the fast processing of sensory stimuli.

## Results

### Modular novelty-familiarity coding

In this paper we consider hierarchical coding of visual stimuli where at each level of the hierarchy it is possible to infer (‘predict’) the activity at the preceding lower level. Mathematically, vector *f*_*i*_ of neuronal firing rates at level *i* can approximate the firing rate vector *f*_*i*−1_ at the lower level, *f*_*i*−1_ ≈ *ϕ*(*U*_*i*_ *f*_*i*_), where *U*_*i*_ is a linear transform and *ϕ* the generative function, a possibly nonlinear function applied component-wise to the linear combinations of neuronal activities at level *i*. To enforce information extraction at each level, complexity constraints are imposed on *f*_*i*_, e.g., that *f*_*i*_ be of lower dimension than *f*_*i*−1_. The approximation quality is measured by a quadratic error,

$$\begin{array}{c} E_{i}=\frac{1}{2}{∥f_{i-1}-\phi \left(U_{i}f_{i}\right)∥}^{2}\;, \end{array}$$ (1)

where for the first level (*i* = 1) the input activity represents the image, *f*_0_ = *I*. Within the classical predictive coding framework [7], the total error function *E*_1_+*E*_2_+⋯+*E*_*L*_ is minimized across *L* levels, with the consequence that the activity vector *f*_*i*_ depends on both the activities of the lower *and* higher levels.

Here, we suggest a modular hierarchical coding which assumes that at each level *i* the corresponding error function *E*_*i*_ is minimized independently of the representation at the higher level (Fig 1). The minimization is achieved both on the time scale of the fast neuronal dynamics and that of the slow synaptic plasticity. For the neuronal dynamics this amounts to calculating the gradient of *E*_*i*_ with respect to the neuronal firing rates *f*_*i*_ at each level separately, and equating the negative of this gradient to the temporal derivative of *f*_*i*_. Assuming that *ϕ* is the identity function, *f*_*i*_ then evolves as

$$\begin{array}{c} \tau {\dot{f}}_{i}=-\frac{\partial E_{i}}{\partial f_{i}}=U_{i}^{T}\left(f_{i-1}-U_{i}f_{i}\right)\;, \end{array}$$ (2)

with some time constant *τ* and $U_{i}^{T}$ being the transpose of matrix *U*_*i*_. As we argue below, nonlinear functions *ϕ* different from threshold-linear would require non-local neuronal processing rendering the corresponding generative model biologically unlikely, at least in the absence of non-monotonic gain modulation.

To make the dynamics Eq (2) neuronally plausible we introduce auxiliary ‘*novelty*’ neurons which represent the difference *n*_*i*_ = *f*_*i*−1_ − *U*_*i*_ *f*_*i*_. Note that this difference expresses a prediction error, i.e., the residual activity in the lower-level neurons *f*_*i*−1_ that cannot be ‘predicted’ by the higher-level neuronal activities *f*_*i*_. In our interpretation, this difference is calculated by recurrent connections within the upper layer *i*, without assuming top-down connections (Fig 2a). Since in reality and in our model the reconstruction is learned based on repeated stimulus presentation, the *f*_*i*_ neurons encode the lower-level activity by exploiting the statistics of all images presented, and hence we refer to the *f*_*i*_’s as ‘*familiarity*’ neurons (also called ‘prediction’ or ‘representation’ neurons—cf. [21]). Since due to the above definition the activity of novelty neurons tracks the prediction errors instantaneously, their neuronal time constant must be short compared to that of the *f*_*i*_ neurons. This dynamical constraint can be taken into account by introducing a small leak term −*ϵf*_*i*_, yielding a long integration time constant for *f*_*i*_ as compared to the dynamics of *n*_*i*_. The leak term can also be considered as additional constraint that keeps the overall activity of the *f*_*i*_ neurons low,

$$\begin{array}{c} E_{i}=\frac{1}{2}{∥I-U_{i}f_{i}∥}^{2}+\frac{\epsilon }{2}\sum_{k=1}^{N_{i}}{\left(f_{i}\right)}_{k}^{2}\;. \end{array}$$ (3)

For biological realism—and to allow for nonlinear computational properties (see [22–24] and below)—we truncate the firing rates of the *f*_*i*_ neurons at 0 whenever they would become negative otherwise. The overall neuronal dynamics minimizing the individual error functions *E*_*i*_ then becomes

$$\begin{array}{c} \begin{array}{ccc} \tau_{f}\;{\dot{f}}_{i} & = & -\epsilon f_{i}+V_{i}n_{i}\;,\;\;\text{constrained}\;\text{to}\;\;f_{i}\geq 0\\ \tau_{n}\;{\dot{n}}_{i} & = & -n_{i}+f_{i-1}-U_{i}f_{i}\;, \end{array} \end{array}$$ (4)

where *U*_*i*_ represents the matrix of synaptic weights from the *f*_*i*_ to the *n*_*i*_ neurons, and its approximate transpose $V_{i}\approx U_{i}^{T}$ the weights from the *n*_*i*_ to the *f*_*i*_ neurons within level *i* (similar, but not the same initial values—see Methods—are more realistic biologically and the transposed update rule Eq (6) lead to *V*_*i*_ approaching $U_{i}^{T}$ as learning progresses). Similar neuronal dynamics applied to a single layer with the whole image as each neuron’s receptive field was introduced in [22].

The same quadratic constraint in Eq (3) also mimics the effect of the missing top-down connections that would introduce a quadratic penalty term on the components not represented by the upper level (Eq (S.7) in S1 Supporting Information). In the cross-level predictive coding scheme, the top-down connections would selectively suppress components that provide less information for the coarse-grained representation at the higher level. But in doing so, the lower-level network will only converge to a steady state when the higher-level network does. This deteriorates the convergence time for the lower level towards that of the higher level, which itself can only extract the relevant information when the lower level dynamics is near relaxation. The dynamics Eq (4), instead, is faster as it does not depend on the more global *f*_*i*+1_ activity.

### Anatomical versus effective connectivity

The dynamics in Eq (4) describes a layered hierarchy of mutually connected familiarity and novelty neurons *f*_*i*_ and *n*_*i*_, respectively, which could be embedded in the visual system. Starting with an image $f_{0}=\tilde{I}$ preprocessed by the lateral geniculate nucleus (LGN), novelty neurons *n*_*i*_ receive feedforward input from familiarity neurons *f*_*i*−1_ of the lower level as well as input from familiarity neurons *f*_*i*_ of the same level. These latter also receive input from novelty neurons *n*_*i*_ of the same level, and are thus embedded in a recurrent network within level *i* (Fig 2a). However, in the steady state we can express the activity of both the familiarity and novelty neurons as a function of input from the previous level: from Eq (2) with ${\dot{f}}_{i}=0$ we obtain $f_{i}=U_{i}^{+}f_{i-1}$, where $U_{i}^{+}$ is the pseudoinverse of *U*_*i*_. Plugging this into the second equation of Eq (4) while setting ${\dot{n}}_{i}=0$ yields *n*_*i*_ as a function of *f*_*i*−1_. Hence, while the recurrent anatomical connectivity is expressed by Eq (4) (Fig 2a), the effective connectivity in the steady state becomes purely feedforward (Fig 2b):

$$\begin{array}{c} f_{i}=U_{i}^{+}f_{i-1}\;\;\text{and}\;\;n_{i}=\left(1-U_{i}U_{i}^{+}\right)f_{i-1} \end{array}$$ (5)

This effective connectivity represents the underlying causalities and effectively drives the responses of familiarity and novelty neurons at level *i* by those of familiarity neurons at level *i* − 1, averaged across the time scale *τ*_*f*_ of the network dynamics (on the order of milliseconds). Due to the modularity, the full representational power at a specific level *i* is exploited to optimize the novelty-familiarity representation at that spatial resolution, while also enabling an optimized, parallel readout from the different levels. In contrast, when optimizing only the single sum *E*_1_+*E*_2_+⋯+*E*_*L*_ across all levels as in the classical ‘cross-level’ predictive coding [7], top-down connections from level *i*+1 introduce additional constraints at level *i* and help to reduce the error at level *i*+1, but this is at the expense of a non-optimal lower-level representation (see Eq (S.7) and Fig A in S1 Supporting Information). A compromise that helps to improve the prediction error at the next level *i*+1 while retaining the full representational power at level *i* is to introduce a quadratic penalty term on the familiarity neurons at level *i* that we used to derive the dynamics for *f*_*i*_ (Eqs (3) and (4)).

### Synaptic plasticity and hierarchical PCA

Learning was implemented in our model by further minimizing each individual error function *E*_*i*_ with respect to the synaptic connectivity matrix *U*_*i*_ on the slow time scale of stimulus presentation. While the input to the first level is clamped at the currently presented image $f_{0}=\tilde{I}$, the neuronal dynamics is relaxed across the levels until it reaches a steady state, and *U*_*i*_ is then updated by gradient descent on the error function *E*_*i*_ (Eq (1)) with respect to synaptic weight parameters *U*_*i*_. Subject to a spatial locality constraint ensuring that outside a small area of the RF the connectivity is always 0, the weight update is

$$\begin{array}{c} \Delta U_{i}=-\eta \;\frac{\partial E_{i}}{\partial U_{i}}=\eta \;\left(f_{i-1}-U_{i}f_{i}^{*}\right)\;{\left(f_{i}^{*}\right)}^{T}=\eta \;n_{i}^{*}\;{\left(f_{i}^{*}\right)}^{T}\; \end{array}$$ (6)

with some learning rate *η*. Here $f_{i}^{*}$ and $n_{i}^{*}$ are steady-state neuronal activities after network relaxation. Notice that this synaptic update rule has the Hebbian form of postsynaptic activity times presynaptic activity. Similarly, the synaptic updates from the novelty to the familiarity neurons take the Hebbian form

$$\begin{array}{c} \Delta V_{i}=\eta \;f_{i}\;{\left(n_{i}\right)}^{T}\; \end{array}$$ (7)

**Fig 3:**
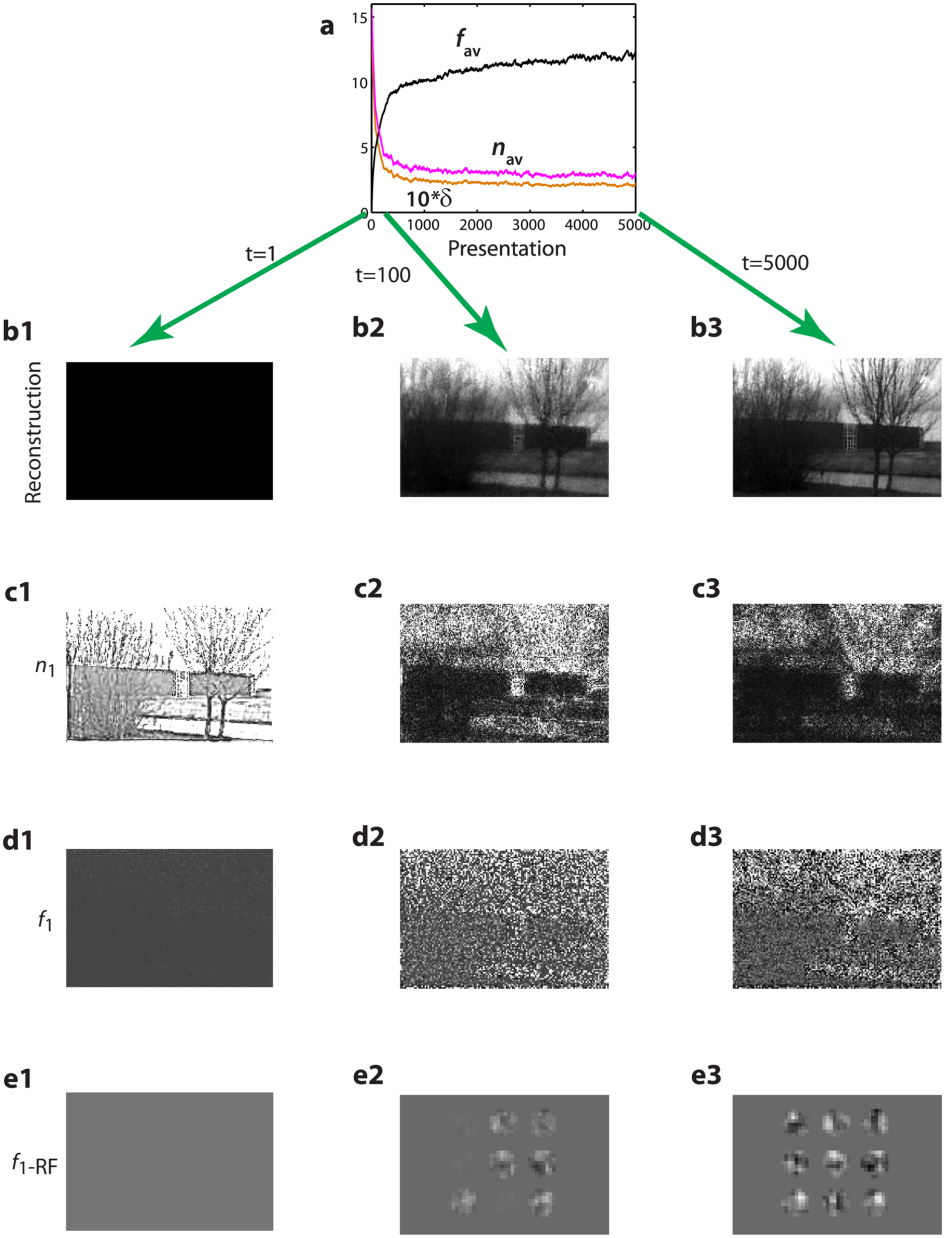
Local recurrent connectivity emerging from unsupervised learning improves reconstruction quality. **a** Evolution of reconstruction error (*δ*, golden, averaged across 50 consecutive presentations) during 5000 random presentations from a set of 1000 training images. Average activity of level-1 novelty neurons (*n*_av_, magenta) mirrors decrease in *δ*. Initial sharp decrease in *n*_1_ activity is explained by average activity increase of level-1 familiarity neurons (*f*_av_, black), which quickly learn to extract the most dominant component, mean local brightness (cf. d2). **b1–b3** Reconstruction of a single novel image (not used for training) after 0, 100, and 5000 presentations based on *f*_1_ activities. **c1–c3** Activity of all novelty neurons *n*_1_ (same number of neurons as pixels in image), showing the reduction of local novelty with decreasing reconstruction error. **d1–d3** Corresponding activities of *f*_1_-neurons in response to the novel image. Image representation is gradually refined, despite the image not having been presented to the network. **e1–e3** Evolution of overlapping receptive fields (RFs, rows of *V*_1_), shown separated for visualization, of nine representative nearest-neighbor familiarity neurons.

In the present case of modular hierarchical coding (but not for the cross-level predictive coding [7]), this architecture performs a hierarchical principal-component analysis (PCA) of the image. PCA is known to minimize the mean squared error ([25, 26]; also demonstrated explicitly in S1 Supporting Information, Section S.II). The familiarity neurons’ activities *f*_*i*_ represent the principal components of the activities *f*_*i*−1_ of the lower-level familiarity neurons by virtue of the effective feedforward connectivity (Eq (5)). But unlike in the previous work [22, 25, 26], the receptive field (RF) of a single *f*_*i*_ neuron is limited to a small area and does not span the whole image. Different components are extracted on the RF overlaps. These components jointly span the space of the first principal modes on the overlaps (cf. Fig 3e and S1 Supporting Information, Subsection S.II). Yet, the PCA property does not imply that the image analysis is linear. Due to the thresholded transfer functions, neurons with overlapping or neighboring RFs may nonlinearly interact to minimize the prediction error on the entire set of images (see Subsection on endstopping below).

### Development of level-1 recurrent connectivity

To track the progress of learning, we considered the evolution of the reconstruction error on a training set of 1000 natural images and the average steady-state activities at the first level. As expected, the average activity *f*_av_ across the familiarity neurons *f*_1_ increases while the average activity *n*_av_ across the novelty neurons *n*_1_ decreases with repeated presentations of the images (Fig 3a, black and magenta curves, respectively). At the same time, the reconstruction of the LGN-preprocessed image *f*_0_ based on the activity of familiarity neurons *f*_1_, *f*_0_ ≈ *U*_1_ *f*_1_, becomes more accurate as expressed by the error curve (Fig 3a, golden) and the example reconstructions (Fig 3b). The topographic representation of the neuronal activities shows that during the learning process the contrast among the novelty neurons decreases (Fig 3c1–3c3) while among the familiarity neurons it increases (Fig 3d1–3d3). Learning transforms novelty into familiarity while keeping the original information (as expressed by *f*_0_ = *U*_1_ *f*_1_+*n*_1_).

Inspection of the receptive fields (RFs) of familiarity neurons *f*_1_, as expressed by the vector of synaptic input strengths from *n*_1_ neurons (rows of *V*_1_), shows RFs composed of patches of excitation and inhibition (Fig 3e). When combined to jointly cover the input space, they form the first principal components of the correlation matrix of the inputs (S1 Supporting Information, Subsection S.II)). An additional sparseness constraint in the energy functional (Eq (1)), e.g., an additional penalty term for the norm of *f*_*i*_ or *U*_*i*_, see S1 Supporting Information), may force the weight vectors to become orthogonal, while the RFs become more Gabor-like, similar to the ones observed biologically [27–29]. Yet, as the characterization of the RFs depends on the choice of stimuli [30–32], we did not intend to reproduce specific RF shapes.

After learning, we assessed the learning quality on a set of 200 novel images from the same library but never presented to the network before. The generalization quality is very good: the average reconstruction error was less than 5% larger relative to that for the images used in learning. Note that a specific image was only presented approximately 5 times (in each of the 5000 presentations, an image was randomly selected out of the 1000 images) during the learning process, and so learning of the local image structure is based on the statistics of all the 1000 images.

### Hierarchical novelty-familiarity representation

**Fig 4:**
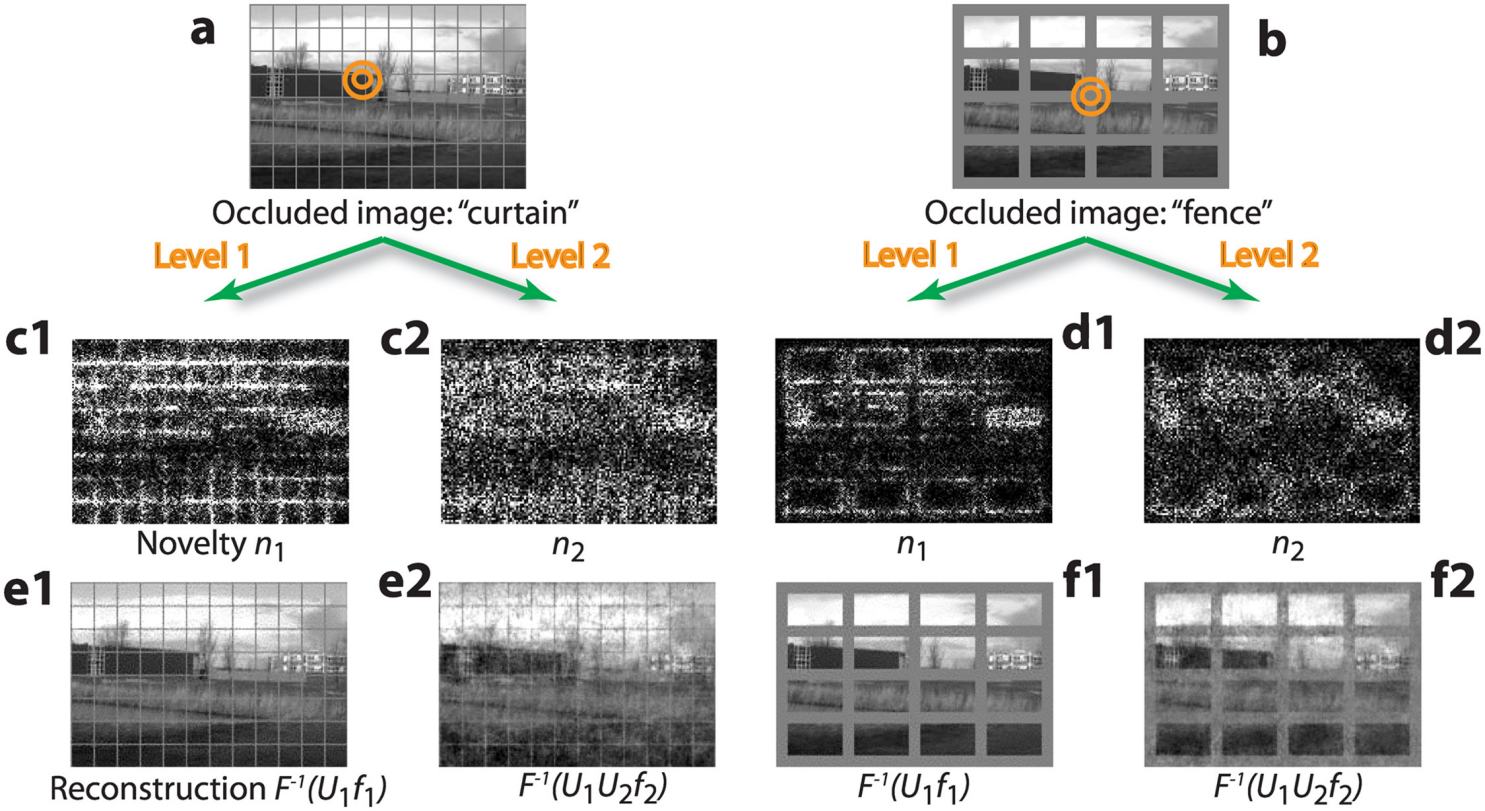
Novelty detection and familiarity information fill-in at different spatial scales. A familiar image is either occluded by a narrow, 1-pixel-wide ‘curtain’ (**a**) or by a 7-pixel-wide ‘fence’ (**b**). Circles represent the RFs of level-1 (smaller circle) and level-2 neurons, respectively. Novelty neurons at level 1 are activated by the fine curtain (**c1**), while at level 2, curtain information is filtered out (**c2**). For the fence, novelty at level 1 is only detected at some edges (**d1**), but novelty of the wide fence bars is detected at level 2 (**d2**). Reconstruction of the image (LGN activity) based on the level-1 and level-2 familiarity neurons for the curtain (**e1, 2**) and the fence occlusions (**f1, 2**) shows how the occluded parts of the image are filled in despite the reduced number of neurons.

To illustrate how the image representation is decomposed into familiarity and novelty signals at different spatial scales, we superimposed a grid of two different line widths onto an image not used for learning (Fig 4a and 4b). Neither grid is a typical feature of natural images, and hence they are detected as novel. However, because of the different line widths, the narrow ‘curtain’ is mainly detected at level 1 (white grid in Fig 4c1, reflecting activity of *n*_1_-neurons), while the area of the wide ‘fence’ is mainly detected at level 2 (white grid in Fig 4d2, reflecting activity of *n*_2_-neurons). The wide bars of the fence are recognized as statistically familiar at level 1 because a level-1 RF is covered by a uniform bar of the fence (narrow circle in Fig 4b), and the uniform brightness as a zero-order principal component can be easily reconstructed by the familiarity neurons. At this first level, only edges along the bars of the fence are partially detected by the novelty neurons (Fig 4d1).

Novelty is a local phenomenon restricted to the receptive field. Where novelty is detected, the original image can be partially reconstructed using surrounding familiarity neurons through lateral connections. In fact, image reconstruction based on the familiarity neurons alone partially succeeds to retouch away the narrow curtain at level 1 (Fig 4e1), and both the fence and the curtain at level 2 (Fig 4e2 and 4f2). As the accurate image reconstructions from the fewer level-2 familiarity neurons show, familiarity information is still available at that level, despite the fact that input to level 2 is only fed into level-2 novelty neurons. This is possible because familiarity neurons (at level 2) continuously integrate incoming information from novelty neurons, until the activity in the novelty neurons cannot be explained anymore by the familiarity neurons (cf. Eq (4)).

**Fig 5:**
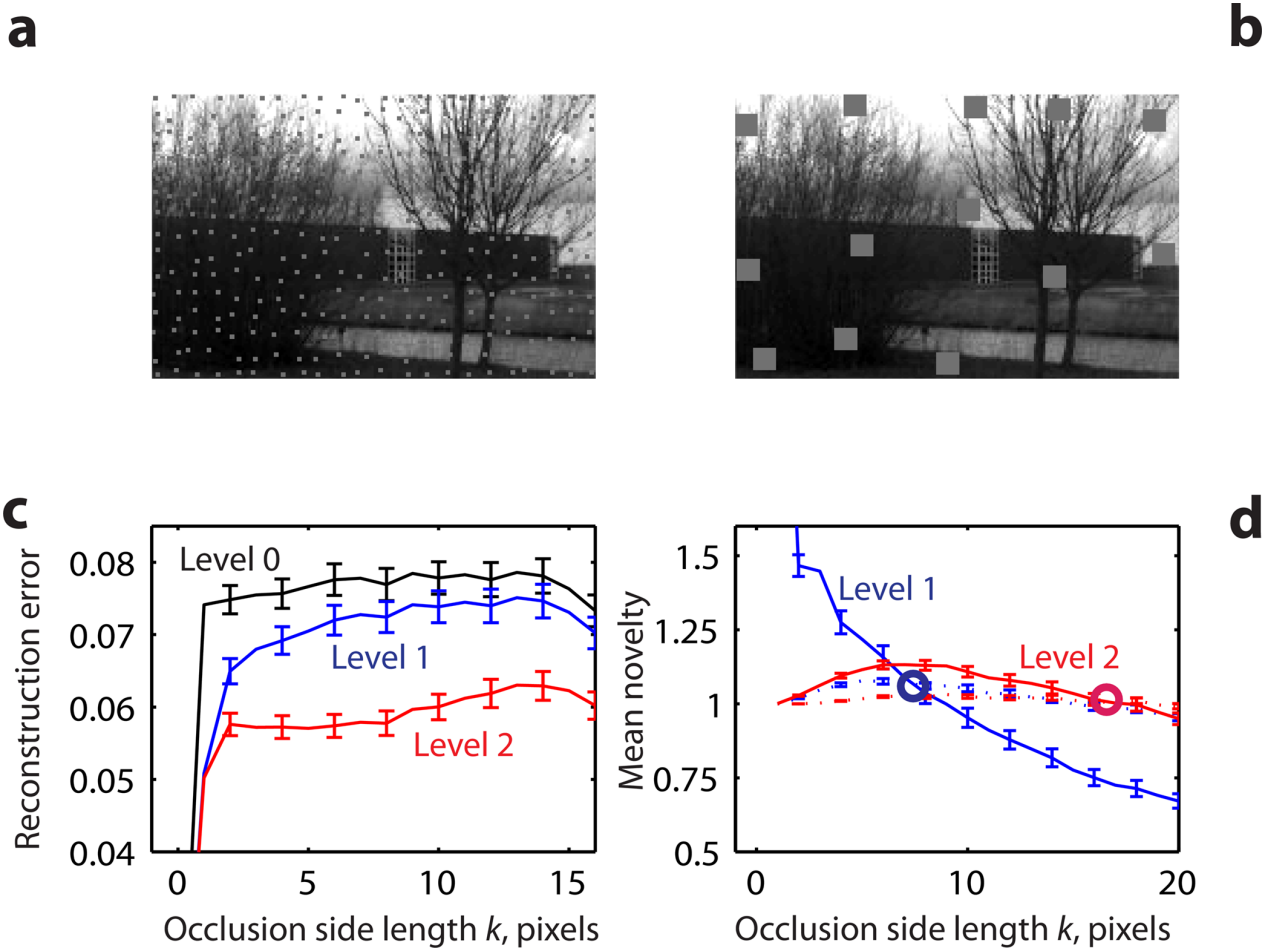
Reconstruction and novelty as a function of occlusion patch size *k*. **a, b** Example of a novel image with occlusion patches (grey squares) of side length *k* = 1 (**a**) and 10 pixels (**b**). For each patch size, the total occluded area is 5% of the image area. **c** Reconstructions of occluded and non-occluded images progressively become more similar across levels. Average distance *δ* (across 200 novel images) between the non-occluded original image *I* and the same image $\hat{I}$ occluded by the patches (black curve, ‘level 0’), and between the reconstructions of those images *F* ^−1^(*I*) and $F^{-1}\left(\hat{I}\right)$ based on the familiarity neurons at level 1 (blue) and level 2 (red), respectively. **d** Average activity of level-1 (blue) and level-2 (red) novelty cells over the occluded (solid) and non-occluded image areas (dotted curves). Novelty neurons in the occluded parts of the image are more active than novelty neurons in the non-occluded parts of the image as long as the patch size *k* is smaller than the RF diameter (8 and 18 pixels for levels 1 and 2—cf. blue and red circles—respectively).

The size of an occlusion that can be correctly reconstructed depends on how small it is compared to the RFs of the corresponding familiarity neurons. To investigate how the reconstruction quality at the first two levels improves as a function of the occlusion size, we consider randomly scattered square-shaped occlusions of the same total area (5% of the image area, Fig 5a and 5b). Reconstruction quality is assessed based on 200 novel images from the same library, but again not part of the training set. As expected, level-1 and level-2 familiarity neurons carry original image information even for occluded patches. The distance between the reconstructions of occluded and non-occluded images is much less than that between the non-occluded and occluded images themselves for small occlusion sizes (*k* = 0 corresponds to no occlusion and, correspondingly, all curves start at 0 for *k* = 0). This suggests that for small occlusion sizes despite the occlusions, it is the original, non-occluded, image that is reconstructed.

The activities of the novelty neurons on the occluded and non-occluded areas represent a biologically feasible measure of reconstruction errors in the respective image areas. Level-1 novelty neurons’ mean activity over the occluded areas (Fig 5d, decreasing solid blue curve) is highest for the smallest occlusion patch size *k* and diminishes progressively with increasing *k*. It equals the non-occluded neurons’ activity (Fig 5d, dotted blue curve) at approximately *k* = 8 pixels (the diameter of a single level-1 RF), and then continues to decrease, indicating that uniform occlusions larger than the size of a single RF are indeed the most familiar and easiest to reconstruct. For level-2 novelty cells, the occluded (Fig 5d, solid red curve) and non-occluded (dotted red curve) mean activities become equal at approximately the size of the level-2 RF (diameter ≈18), as expected. The solid red curve, unlike the blue one, begins at 1 for the smallest occlusion of size *k* = 1 (and remains non-monotone for small occlusion sizes). This is a consequence of the natural definition of the RFs of level-2 novelty neurons as equal to those of the corresponding level-1 familiarity neuron into which the occlusions fall. E.g., for *k* = 1 the 5% total occlusion translates into approximately 1 pixel of occlusion for every 4.5 pixels of the image and hence, on average, every level-2 novelty neuron’s RF contains a pixel of the occlusion when *k* = 1.

### Non-classical RF property of endstopping results from within-layer connectivity

To test for the non-classical RF property of endstopping, we stimulated each level-1 familiarity neuron separately by a uniform bar extending horizontally across the whole neuron’s RF and vertically across 40% of the RF diameter. The bar was approximately 5 times brighter (similarly to [7]) than the background, whose brightness was equal to the average over all training images. The equilibrium activity of each level-1 familiarity cell’s response to a horizontal bar was then recorded and the 20% of the neurons with the highest equilibrium activities were designated bar detectors. These detectors were then stimulated, again one at a time, by bars of the same width (equal to the RF diameter) and height varying systematically from 0 to 3 RF diameters. The activity deviations of all the novelty cells within each bar detector’s RF from their rest values, averaged over each bar detector’s RF, were computed for each height.

**Fig 6:**
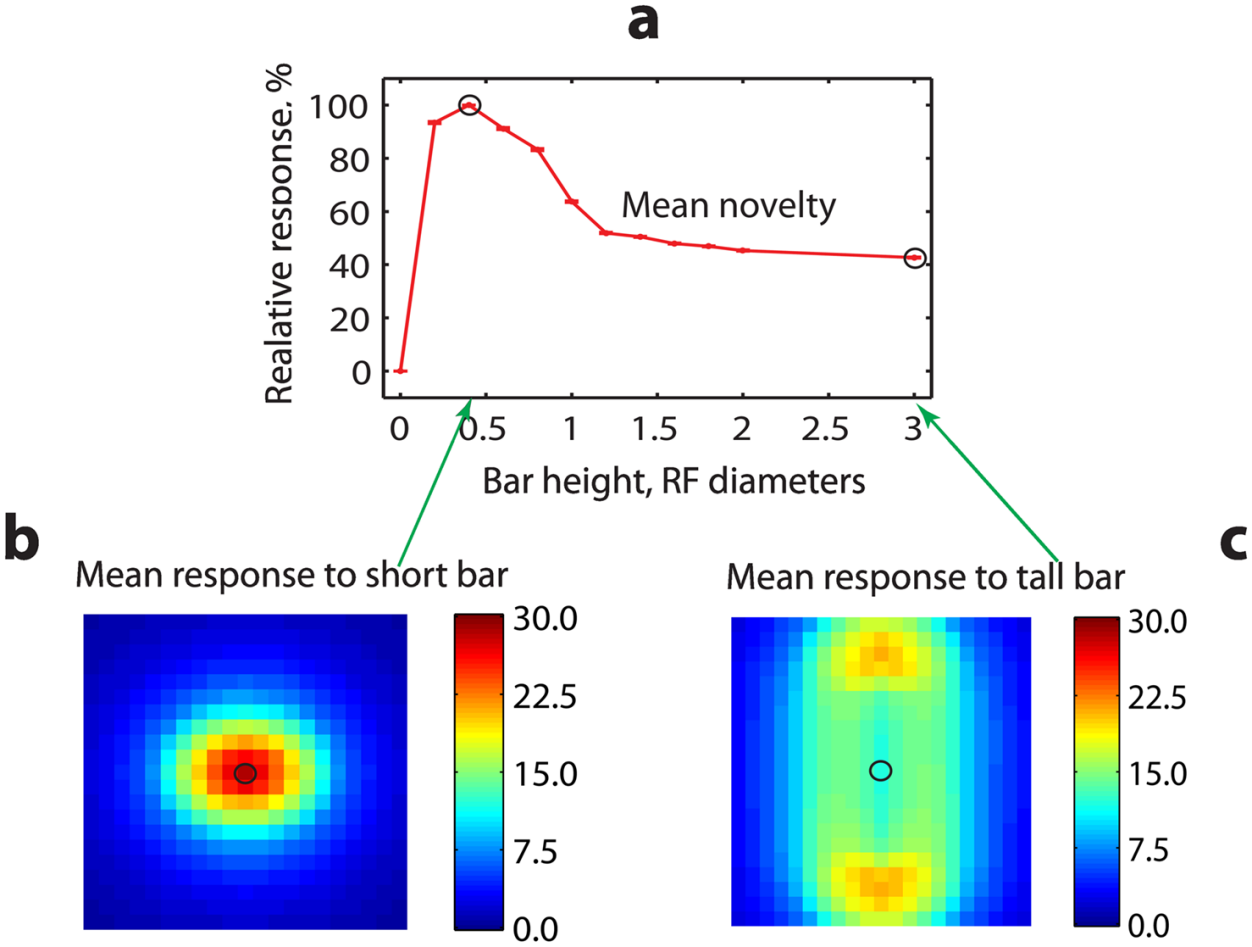
Non-classical effect of endstopping is present in the effectively feedforward architecture of modular predictive coding. **a** Activity of level-1 novelty neurons averaged across the RF of bar-detecting level-1 familiarity neurons decreases as bar height exceeds approximately one-half of the RF diameter. Activity is shown relative to baseline activity for the short bar of 0.4 RF diameter and five times brighter than the background. Black circles indicate the responses of bar detectors relative to the baseline for short and tall bars in panels b and c, respectively. **b** Average activities of level-1 novelty neurons in a neighborhood of 3.5-by-3.5 RF diameters around each bar-detector in response to the short bar. Central dark red square (black circle) represents the average activity of all novelty cells within the RF of a bar detector, further averaged across all bar detectors. The same double averaging was applied to the novelty neurons within the RFs of the neighboring familiarity neurons. While these RFs are highly overlapping, they are displayed here side-by-side. **c** Same as in panel b, but in response to a tall bar of height 3 (instead of 0.4) RF diameters. The averaged activity of novelty neurons (representing the local reconstruction error) within the bar-detectors RF (black circle) is lower than that for the short bar (compare black circles) and for the same reason also lower than that recorded at the ends of the bar (top and bottom yellow patches).

The relative response of the novelty neurons decreased when the bar height extended beyond 0.4 RF diameter (Fig 6a) indicating cooperative effects between the familiarity cells with overlapping RFs. To illustrate the typical behavior of novelty neurons, we also averaged novelty activity across RFs of the familiarity neurons in a fixed-size neighborhood of approximately 3.5-by-3.5 RF diameters around each bar detector. The responses to the short stimulus of height 0.4 RF diameter (Fig 6b) and the tallest stimulus of height 3 RF diameters (Fig 6c) show that for the tall stimulus, despite the same brightness, the activity is considerably lower and more uniform. The reduction of novelty activity in the bar center with increasing bar height reveals an effective polysynaptic inhibitory effect of the neighboring neurons on the bar detectors, consistent with the endstopping behavior of bar-detecting neurons in the primary visual cortex arising from lateral connectivity [33–35]. The comparison between the response to the short bar (Fig 6b) and that to the tall bar (that itself can be considered as a juxtaposition of 3 vertically aligned bars of height 1) reveals the nonlinear inhibitory processing due to the thresholding of the *f*_1_ activities. In fact, the response to the tall bar (Fig 6c) is much weaker than the sum of the responses to the bars of height 1 would be.

### Nonlinear predictive coding is neuronally implementable for threshold-linear transfer functions only

Predictive coding has been considered within a general framework of optimization principles allowing for nonlinear feature extraction [16, 21, 36]. In striving for a neuronal implementation of such nonlinearities, however, we find below that it is essentially only the linear neuron model with the quadratic error function that can be implemented by neurons using locally available information. Yet, threshold-linear neurons allow the predictive coding model to remain linear while enabling nonlinear feature extraction. Given the representation of the unconstrained activities *f* by a difference of threshold-linear ‘ON’ and ‘OFF’neurons, *f* = ⌊*f*⌋ − ⌊−*f*⌋, another readout neuron may easily extract the sum ⌊*f*⌋ + ⌊−*f*⌋. Qualitatively, the latter operation is similar to taking the square of the linear filter output, *f* ^2^, which was shown to lead to phase-invariant receptive fields of complex [37] or motion-selective cells [38]. Hence, the neuronal implementation of the linear version of modular predictive coding yields the ingredients to explain the ON-OFF simple cells [39] and also complex cells in the primary visual cortex (V1) as they arise in nonlinear optimization models [40, 41].

#### Implementation of positive transfer functions

As everywhere in this paper, the neuronal activities *f*_*i*_ were kept positive by truncating them at 0 should they become negative. We also explored the option of a non-negative prediction error. To achieve this, we applied a shift to the *n*-activities by a vector *n*_∘_ with equal and positive components (large enough so that the *n*_*i*_ became nonnegative at all times) and then subtracted this shift again to calculate ${\dot{f}}_{i}$ and the weight changes as if *n*_*i*_ had not been offset. Hence, instead of Eqs (4), (6) and (7) we consider

$$\begin{array}{c} \begin{array}{ccc} \tau_{f}{\dot{f}}_{i} & = & -\epsilon f_{i}+V_{i}n_{i}-b_{i}\;,\;\text{constrained}\;\text{to}\;f_{i}\geq 0\\ \tau_{n}\;{\dot{n}}_{i} & = & -n_{i}+n_{\circ }+f_{i-1}-U_{i}f_{i} \end{array} \end{array}$$ (8)

$$\begin{array}{c} \begin{array}{ccc} \;\Delta U_{i} & = & \eta \;\left(n_{i}^{*}-n_{\circ }\right)\;{\left(f_{i}^{*}\right)}^{T}\\ \Delta V_{i} & = & \eta \;f_{i}^{*}\;{\left(n_{i}^{*}-n_{\circ }\right)}^{T}\\ \Delta b_{i} & = & \eta^{b}f_{i}^{*} \end{array} \end{array}$$ (9)

Here, again, $f_{i}^{*}$ and $n_{i}^{*}$ are the steady-state neuronal activities after network relaxation, while *η* and *η* ^*b*^ represent learning rates. The bias *b*_*i*_ must converge towards *V*_*i*_ *n*_∘_ due to the steady state conditions 〈*n*_*i*_〉 = *n*_∘_, which express that the prediction errors *n*_*i*_ − *n*_∘_ are balanced around 0. This is the case because $f_{i}^{*}$ on the right-hand side of the *b*_*i*_-update equation is a locally available approximation to the negative gradient of ${\left(V_{i}n_{i}^{*}-b_{i}\right)}^{2}$ with respect to *b*_*i*_.

#### Nonlinear generative functions do not have a neuronal implementation

When considering a nonlinear generative function *ϕ*, the gradients of Eq (1) with respect to *f*_*i*_ and *U*_*i*_ become, instead of Eqs (2) and (6),

$$\begin{array}{c} \tau {\dot{f}}_{i}=U_{i}^{T}\;(\left(f_{i-1}-\phi \left(U_{i}f_{i}\right)\right).*\phi^{\prime }\left(U_{i}f_{i}\right)) \end{array}$$ (10)

$$\begin{array}{c} \Delta U_{i}=\eta \;(\left(f_{i-1}^{*}-\phi \left(U_{i}f_{i}^{*}\right)\right).*\phi^{\prime }\left(U_{i}f_{i}^{*}\right)){\left(f_{i}^{*}\right)}^{T}\;, \end{array}$$ (11)

where.* is the componentwise multiplication, *η* is a positive learning rate and f* is the steady-state activity.

Any nonlinear generative function *ϕ* that is different from threshold-linear introduces a nontrivial multiplicative modulation of the synaptic input to the *f*_*i*_-neurons as expressed by the pointwise product in Eq (10). The same product also arises in updating the weight matrix *U*_*i*_ in Eq (11). By again considering the difference in the above equations as auxiliary neuronal quantities, *n*_*i*_ = *f*_*i*−1_ − *ϕ*(*U*_*i*_ *f*_*i*_), the modulation by *ϕ*′(*U*_*i*_ *f*_*i*_) even becomes non-local. In fact, the input *f*_*i*_ weighted by the synaptic strengths, *U*_*i*_ *f*_*i*_, would then become the input to a *n*_*i*_ neuron and so it is not locally available at the site of the *f*_*i*_ neuron (without assuming a specific rewiring and duplication of synaptic weights).

Alternatively, one may postulate a neuron with highly non-monotonic interactions among different synaptic inputs as expressed by the steady state equation of that neuron, *n*_*i*_ = (*f*_*i*−1_ − *ϕ*(*U*_*i*_ *f*_*i*_)).*ϕ′(*U*_*i*_ *f*_*i*_). This would imply, on the one hand, that some part of the synaptic current *U*_*i*_ *f*_*i*_ is nonlinearly added to the other input *f*_*i*−1_, while on the other hand, the total postsynaptic current is multiplicatively modulated by the derivative *ϕ*′(*U*_*i*_ *f*_*i*_). Although multiplicative gain modulation, say by some dendritic input, is possible [42], this modulation would be non-monotonic since *ϕ*′ (for a sigmoidal function *ϕ*) is 0 for both small and high values.

#### A possible non-quadratic error function

Further investigating other possible nonlinearities in the optimization problem at hand, we also considered the error function

$$\begin{array}{c} E_{U_{i}}\left(f_{i},f_{i-1}\right)=\Psi (f_{i-1}-U_{i}f_{i})\; \end{array}$$

with $\Psi \left(x\right)=\frac{1}{\alpha^{2}}\log\cosh\alpha x\;$ applied component-wise. For small *x* this error is quadratic, $\Psi \left(x\right)\approx \frac{1}{2}x^{2}$, and for large *x* it is linear, $\Psi \left(x\right)\approx \frac{1}{\alpha }\left|x\right|$. We can combine this nonlinearity with the rectification of *f*_*i*_ and the upwards shift of *n*_*i*_. In the neuronal and synaptic dynamics specified by Eq (8) only the second line then changes to

$$\begin{array}{ccc} \tau_{s}\;{\dot{n}}_{i} & = & -n_{i}+n_{\circ }+\Psi^{\prime }\left(f_{i-1}-U_{i}f_{i}\right)\;, \end{array}$$

where $\Psi^{\prime }\left(x\right)=\frac{1}{\alpha }\tanh\;\alpha x\approx x$ for small *x* is a standard sigmoidal nonlinearity often considered in modeling a saturating neuronal transfer function. In computational terms, the nonlinearity *Ψ* tends to alleviate the effects of statistical outliers in input stimuli. Our simulations (with optimized parameter *α* = 4, not shown), however, reveal that these benefits are rather humble. Hence, all of the results presented in this paper are for the simpler, more transparent model described by Eqs (8) and (9).

## Discussion

We have reconsidered predictive coding as an organization principle of the visual cortex. While in the original work the anatomical feedforward propagation of information from novelty neurons has been emphasized [7, 10, 11], we show that in functional terms it is actually the familiarity, not the novelty, information that is fed to the next level. This apparent conundrum arises because the only anatomical connections from a lower to a higher level project from the lower-level familiarity neurons into the higher-level novelty neurons (Fig 2). However, at the higher level, it is again the familiarity information that is extracted from the feedforward input, although now at a coarser resolution. The network dynamics continuously separates familiarity from the novelty information while simultaneously building up both representations.

The modular predictive coding scheme that we are proposing here assumes that on the fast recognition time scale features are only extracted via level-specific recurrent circuitry, not via top-down projections (Fig 1). This modularity is a compromise between the fully backward-connected original coding scheme [7] and a purely feedforward hierarchy without lateral connectivity [1]. Viewing stimulus representation as being modular without top-down feedback has distinct computational advantages. First, the network computation can be understood as a hierarchical principal component analysis with increasing receptive field sizes. Second, it reduces relaxation times and hence the time for feature recognition at various spatial resolutions that has been shown to be as fast as 30–100ms [17–19]. Third, at each level the full representational power of all neurons is used to optimally extract the information at the corresponding level of granularity. Correspondingly, stimulus compression is achieved solely by limiting the number of prediction neurons, not by shunting the activity by top-down inputs from the higher level.

While our model does not include feedback projections from higher levels, we do not suggest that they would not be of functional use. The ubiquitous top-down connections [43, 44] may have an important functional role in attention gating [45], in memory retrieval initiated from higher cortical areas [46], or in gating synaptic plasticity by a modulatory input from other areas [47–49]. However, we do propose that such connections may not be essential in shaping the stimulus representations on the short-term time scale of the neuronal dynamics. In fact, assuming that the activity of the novelty and familiarity neurons can be read out from all levels of the hierarchy, additional top-down projections from within the predictive coding network cannot provide new information about the stimulus. Learning, on the other hand, provides a helpful means to extract the relevant information by separating novelty from familiarity. While learning strengthens familiarity and makes novelty more salient, the full information remains accessible in the combined novelty-familiarity representation. When restricted to the familiarity neurons, however, the original image is re-represented at each new level in a more compressed form by filling in the within-level predictions of progressively increasing size (Figs 3–5).

Despite the lack of top-down projections—and different from [20] where additional multiplicative and divisive nonlinearities were introduced—our model explains the emergence of non-classical receptive field properties via lateral connectivity alone. Such results are in line with recent experimental findings showing that RFs of V1 cells cannot be defined without considering their spatiotemporal context [30–32, 50]. In our case, the context sensitivity is facilitated by the fact that we did not impose a strict sparseness constraint, but instead derived a ‘soft’ quadratic penalty term from the full predictive coding scheme. In contrast to the sparseness constraint that enforces zero activity, the soft constraint still allows for low activity that may accumulate and shape the non-classical RF properties. Yet, learning still reduces the overall activity within each level and hence leads to a ‘soft sparseness’. This, in addition, arises because learning decreases the prediction error and hence decreases the activity of the numerous novelty neurons.

Considering possible neuronal implementation of predictive coding, we have arrived at severe restrictions on the generative functions prompted by the requirement of compatibility with the current knowledge on neuronal processing. We found that, in essence, the only type of nonlinearity for generative functions that is neuronally implementable is the threshold-linear function, whereas other nonlinearities would require non-local processing. However, use of the threshold-linear transfer function still results in the neuronal processing becoming nonlinear as shown by the non-classical RF properties (Fig 6b and 6c), making the suggested PCA effectively nonlinear. It has been shown that with such a nonlinearity, PCA can extract independent components [23, 24]. We further suggest that the thresholding of the linear transfer function at zero leads to the emergence of ON-cells and OFF-cells [39] that would combine to produce a fully linear response function or to a response function with complex cell properties [41]. How far modular predictive coding as presented here can explain such nonlinear properties of V1 neurons, however, is yet to be analyzed in detail.

## Methods

### Model inputs, structure and fast dynamics

We applied the modular coding scheme to a set of 1000 grey-level images *I* (128-by-192 pixels) from a natural image library [51]. They were passed through localized center-surround filters (Fig 1a and 1b) intended to mimic the combined effects of eye adaptation and LGN processing. From each pixel’s brightness in the image we subtracted 80% of the mean brightness, including that of the pixel itself, in a small circular neighborhood of radius 5 around that pixel. We implemented this filtering for each image *I* by performing a Fast Fourier Transform (FFT) of the image, multiplying it by the FFT of the filter, and then taking the inverse FFT to obtain the filtered image $\tilde{I}=F\left(I\right)$.

The output of the LGN, an image $\tilde{I}$ of the same size as *I*, was fed into the network with dynamics defined in Eq (4), $f_{0}=\tilde{I}$ and two further levels *i* = 1,2. The truncation of *f*_*i*_ at 0 ensured that the activities remained positive. In view of this rectification we doubled the number of the familiarity neurons to maintain the reconstruction quality as compared to unconstrained neurons used, e.g., in [7]. The total number of (threshold-linear) familiarity neurons in the first level thus was 2 times, and in the second level 4 times as small as the number of pixels in the image, leading to a gradually compressed image representation across levels. When the truncation of *f*_*i*_ at 0 was not applied, we obtained very similar results for the significantly higher compression factors of 4 and 16 in the first and second levels, respectively (data not shown). Each filtered image was presented to the network for 5*τ*_*f*_ time units so that the fast dynamics could equilibrate. To keep the dynamics of the novelty neurons fast as compared to one of the familiarity neurons, we set the *n*_*i*_ activities to their equilibrium levels, effectively allowing their instantaneous equilibration and corresponding to *τ*_*n*_ = 0.

We have also carried out extensive simulations with non-instantaneous *n*_*i*_-dynamics and nonnegative *n*_*i*_-values for more biological realism as per Eqs (8) and (9); the results were similar (not shown in this paper).

Other fast-dynamics parameters were as follows: *ϵ* = 0.01, *τ*_*f*_ = 1; integration by the forward Euler method with time step *δt* = 0.03. The matrix of level-1 familiarity cells defining the length of the activity vectors *f*_1_ was 91-by-136 so that the total number of familiarity neurons was one half that of pixels in the image, whereas the number of the level-1 novelty neurons was equal to the total number of pixels in the image, i.e., 24576. The radius of the circular receptive field (RF) within level 1, defining the non-zero row entries for (24’576-by-12’288 dimensional) synaptic matrix *U*_1_, was equal to 4 (yielding ≈50 entries for each row, i.e., pixels in the RF of a familiarity neuron). For level 2, the procedure was exactly the same, except that the feedforward input to level 2 was provided by the *f*_1_-values at the end of the fast dynamics, and these values were not filtered. The size of the second-level familiarity matrix was 64-by-96, and the RF radius defining the row entries of (12’288-by-6’144 dimensional) matrix *U*_2_ was again 4 (which implies that its effective RF radius in terms of the original image is $\approx 9.0=3.5*\sqrt{2}+4$: there will be on average 3.5 distances ($\sqrt{2}$ in terms of original interpixellary distance) between level-1 familiarity cells within the level-2 RF and the level-1 familiarity cells outermost in the level-2 RF will contribute their full radius in terms of the original image, i.e., 4).

### Synaptic plasticity

The entire learning session consisted of presenting the 1000 filtered images, randomly sampled in a total of *T* = 5000 presentations. Following the equilibration of the fast neuronal dynamics during an image presentation, the synaptic weights were updated according to Eqs (6) and (7). The non-zero entries of the weight matrices *U* and *V* (corresponding to the RFs) were initialized independently with a mean of 0 and standard deviation of ≈0.0006 for both levels 1 and 2. The learning rate for *U* was initialized to be $\eta_{0}^{U}=0.0001$ for level 1 and $\eta_{0}^{U}=0.0002$ for level 2. During the course of the image presentations these learning rates were gradually reduced to one-fifth of the original rates according to the schedule *η*_*k*_ = *η*_0_/(1 + 4*k*/*T*) that defined the learning rates at the *k* ^*th*^ presentation (*k* = 1…*T*).

### Performance measures

To quantify how good a reconstruction of an input image familiarity neurons *f*_*i*_ at each level would allow, we evaluated the distance *δ* between the filtered image $\tilde{I}$ (LGN output) and its reconstruction based on the activity of level-1 or level-2 familiarity neurons, $\tilde{I}\approx U_{1}f_{1}$ and $\tilde{I}\approx U_{1}\;U_{2}f_{2}$, respectively. As a distance measure between two images (or between two activity vectors) *a* and *b* we used the Euclidean (*l* ^2^) norm of the difference of normalized images (represented as vectors), *δ*(*a*, *b*) = ‖*a*/‖*a*‖ − *b*/‖*b*‖‖. For visual comparison purposes, we also presented the reconstructed images throughout the paper as inverted back into the original image space (e.g. Fig 1d1 and 1d2). The brain does not need to perform such an explicit reconstruction. Instead, it may differentially use the local familiarity and novelty information at each level for further processing and for obtaining the full original information. In fact, after the relaxation of the neuronal dynamics (Eq (4) with ${\dot{f}}_{i}={\dot{n}}_{i}=0$), the lower-level activity could be fully reconstructed, e.g. *f*_0_ = *U*_1_ *f*_1_+*n*_1_, while the information is segregated into familiarity and novelty representations at the higher level (Fig 1c–1e).

To trace the various quantities during the learning process in Fig 3a, we low-pass filtered these quantities according to $\bar{x}:=\left(1-\lambda \right)\bar{x}+\lambda x$, with *λ* = 0.02, *x* representing the reconstruction error *δ* or the spatial average of neuronal activities *f*_1_ and *n*_1_, respectively, and $\bar{x}$ being the low-pass-filtered version of the same quantity.

The reconstruction error in Fig 5c was calculated using the distance measure *δ*(*a*, *b*) between the reconstructed images *a* and *b* corresponding to cases with and without the occlusion, respectively. This choice highlights the effects of occlusion rather than reconstruction errors present both with and without the occlusion.

## Supporting Information

S1 Supporting Information Modular predictive coding analysis in further detail. (PDF)
