# Supplementary material for: Hierarchical Novelty-Familiarity Representation in the Visual System by Modular Predictive Coding
Source: PLoS One. 2015 Dec 15;10(12):e0144636. doi: 10.1371/journal.pone.0144636 (PMC4682867; doi:10.1371/journal.pone.0144636)
Supplement: S1 Supporting Information — (PDF) [file pone.0144636.s001.pdf]

Supporting Information

for

Hierarchical Novelty-familiarity Representation in the Visual System by Modular Predictive Coding

Boris Vladimirskiy[1,¤a], Robert Urbanczik[1], Walter Senn[1,*]

1 Department of Physiology, University of Bern, Bühlplatz 5, 3012 Bern, Switzerland

¤a Currently at Munich Re UK & Ireland Life, Plantation Place, 30 Fenchurch Street, London EC3M 3AJ, United Kingdom
*** Senn@cns.physio.unibe.ch**

Here we first present the framework of the classical 'cross-level' predictive coding (Section S.I). We show that for a single level, with a readout at the next level but without top-down connectivity, predictive coding performs principal component analysis (Section S.II). We next argue that cross-level predictive coding across 2 levels can essentially be reduced to a single level (Section S.III). To keep the computational benefit of a hierarchical principal component analysis and to reduce processing time throughout the levels, we therefore reduce the cross-level predictive coding to our modular predictive coding scheme (Section S.IV). These sections form the background for Subsection of the main text in which we consider alternative coding schemes and conclude that in terms of a neuronal implementation, all the generative models (e.g., as reviewed in [1]) essentially reduce to the quadratic error function with a linear transfer function.

S.I Classical cross-level predictive coding

In [2], predictive coding was introduced as a hierarchical model of visual processing based on a global objective function summed across the individual components

$$E_i = E_{U_i}(f_i, f_{i-1}) = \tfrac{1}{2}\|f_{i-1} - \phi(U_i f_i)\|^2 \tag{S.1}$$

at each level i of the hierarchy (cf. Eq. (1) in the main text). Here, f_i is the vector of neuronal activations at level i (with 'f' standing for 'familiarity'), and $f_0 = I$ being the input into the system. U_i is a matrix of suitable dimensions with $\phi(U_i f_i)$ representing the prediction of the activity in level $i-1$ based on level-i activity.

Notice that in a Gaussian noise scenario, where each component of the low level activity f_{i-1} is contaminated by an additive Gaussian noise of mean 0 and variance 1, the log-likelihood of the 'data' f_{i-1} given the 'parameters' U_i and f_i is just a constant minus the error function (Eq. (S.1)), i.e., formally $\log P(\, f_{i-1} \,|\, U_i, f_i\,) = C - E_i$, with $C = -N_{i-1}\log(2\pi)/2$ and N_{i-1} the dimension of f_{i-1}. Hence, maximizing the log-likelihood of the lower-level data is equivalent to minimizing the reconstruction error. This links the predictive coding scheme (S.1) to various types of Bayesian modeling [1].

The global objective function according to [2] is just a weighted sum over the levels,

$$E_1 + \cdots + E_L = E(U, f; I) = \sum_{i=1}^{L} E_{U_i}(f_i, f_{i-1})\,. \tag{S.2}$$

Recognition in this system amounts to minimizing $E(U, f; I)$ with respect to f_i for $i > 0$ to obtain

$$E(U; I) = \min_{f_i, i>0} E(U, f; I)\,,$$

and learning amounts to a slow dynamics in U_i minimizing the average $\langle E(U; I)\rangle_I$ of the errors obtained during recognition.

For recognition the relevant gradients defining the temporal evolution df_i/dt of the neuronal activity in the i-th level are

$$\nabla_{f_i} E(U, f; I) = \nabla_{f_i} E_{U_i}(f_i, f_{i-1}) + \nabla_{f_i} E_{U_{i+1}}(f_{i+1}, f_i) \tag{S.3}$$

if $1 \leq i < L$, whereas for $i = L$ the second term in the sum is absent. Hence the gradient in f_i also depends on the activations in level $i + 1$ and this in effect introduces a global coupling of the system. If there are many levels such a global relaxation process becomes difficult to reconcile with the time constraints of visual processing.

S.II Single-level predictive coding performs principle component analysis (PCA)

Since, as per Subsection 2.7 of the main text, only linear generative functions ϕ have a neuronal implementation we set $\phi = \mathrm{id}$ (as also done in [2]). We first consider a single level $L = 1$ so that the global objective function reduces to

$$E_1 = E_{U_1}(f_1, I) = \tfrac{1}{2}\|I - U_1 f_1\|^2$$

Minimizing this with respect to f_1 yields $f_1 = U_1^+ I$, where U_1^+ is the Moore-Penrose pseudo-inverse[1] of U_1. Inserting this in the above inequality yields for $E(U; I) = \frac{1}{2}\|(1 - U_1 U_1^+)I\|^2$. Note that $U_1 U_1^+ = P_{U_1}$ represents the orthononal projection onto the subspace spanned by the columns of U_1. Similarly, $1 - P_{U_1} = P_{U_1^\perp}$ represents the orthonormal projection onto the orthogonal complement of the space spanned by the columns of U_1. We hence obtain $E(U_1; I) = \frac{1}{2}\|P_{U_1^\perp} I\|^2$.

Using the last equation we calculate the expected error across images:

$$\begin{aligned}
\langle E(U_1; I)\rangle_I &= \tfrac{1}{2}\left\langle \left(P_{U_1^\perp} I\right)^T P_{U_1^\perp} I \right\rangle_I \\
&= \tfrac{1}{2}\left\langle \mathrm{Tr}\left(P_{U_1^\perp} I \left(P_{U_1^\perp} I\right)^T\right)\right\rangle_I \\
&= \tfrac{1}{2}\left\langle \mathrm{Tr}\left(P_{U_1^\perp} I I^T \left(P_{U_1^\perp}\right)^T\right)\right\rangle_I \\
&= \tfrac{1}{2}\mathrm{Tr}\left(P_{U_1^\perp} \left\langle I I^T\right\rangle_I\right)\,.
\end{aligned}$$

To arrive at the last line we used the fact that within the argument of the trace operator the three symmetric matrices commute, $\mathrm{Tr}(ABC) = \mathrm{Tr}(ACB)$, and that for any projection P_U one has $P_U P_U^T = P_U^2 = P_U$. Hence, to find the minimum of the above expression across U_1, the columns of $U_1^\perp$ must span the subspace of eigenvectors

[1] If the columns of $U = U_1$ are linearly independent then $U^T U$ is invertible and $U^+ = (U^T U)^{-1} U^T$.

of the correlation matrix $\langle II^T \rangle_I$ with the smallest eigenvalue. Put differently, the columns of the matrix U_1 itself must span the eigenspace corresponding to the largest eigenvalues. If U_1 has N_1 columns and an image I is encoded by N_0 pixels, the dimension of this subspace is $N_0 - N_1$. We then get

$$\min_{U_1} \langle E(U_1; I) \rangle_I = \tfrac{1}{2} \min_{U_1} \operatorname{Tr}\left(P_{U_1^\perp} \langle II^T \rangle_I \right) = \tfrac{1}{2} \sum_{k=N_1+1}^{N_0} \lambda_k^{(0)} \,, \tag{S.4}$$

where $\lambda_k^{(0)}$ are the $N_0 - N_1$ smallest eigenvalues of the correlation matrix $\langle II^T \rangle_I$.

Hence, $U_1 U_1^+ = P_{U_1}$ is the projection onto the first N_1 principal components of the images I, and since in the steady state we have $f_1 = U_1^+ I$, the f_1 must jointly encode these first principal components. Thus, finding U_1 which minimizes the energy function essentially corresponds to doing principal component analysis (PCA). Compared to other PCA algorithms, the present procedure may be more neural but is also less constrained, since, after minimizing in f_1, the relevant object is not U_1 but P_{U_1}. So any two matrices spanning the same subspace become equivalent and, in particular, there is no reason why the U_1 we find should have, say, orthonormal columns. For the same reason there is no scale in the system, any nonzero scalar multiple of U_1 is just as good as U_1.

In many applications of predictive coding, the possible values of U_1 are constrained by the fact that the neurons of f_1 should have limited receptive fields (RFs). In fact, if a RF of a first level neuron covers p_0 pixels in the image, the corresponding column of U_1 will have $N_0 - p_0$ zero entries. However, this does not affect the above analysis, and there the full optimization just has to be replaced by a constrained optimization. This leads to a constrained PCA where the individual neurons jointly represent the eigenspaces of the input. Assuming an n_1-fold overlap of RFs at any position in the image space, the minimization procedure will extract the first n_1 eigenvectors of the $N_0 \times N_0$ correlation matrix $\langle II^T \rangle_I$ averaged across all images I.

S.III Reduction of cross-level to modular predictive coding

Error minimization across neighboring levels

$$E(U, f; I) = E_{U_1}(f_1, I) + E_{U_2}(f_2, f_1) \,.$$

Minimizing $E(U, f; I)$ across f_1 and f_2 will, at the top level, still lead to an optimal f_2 of the form $f_2 = U_2^+ f_1$, whatever f_1 is. As above we calculate

$$\begin{aligned} E_{U_2}(f_2, f_1) &= \tfrac{1}{2}\|f_1 - U_2 f_2\|^2 = \tfrac{1}{2}\|(1 - P_{U_2}) f_1\|^2 \\ &= \tfrac{1}{2}\|P_{U_2^\perp} f_1\|^2 \,. \end{aligned}$$

Note that this reduces the optimization problem across both levels to an optimization within only the first level,

$$E(U, f; I) = \tfrac{1}{2}\|I - U_1 f_1\|^2 + \tfrac{1}{2}\|P_{U_2^\perp} f_1\|^2 \,. \tag{S.5}$$

The reduced optimization problem consists of finding the $N_1 \times N_2$ matrix U_1 (mapping the N_2- to the N_1-dimensional space) and the $(N_1 - N_2)$-dimensional subspace orthogonal to the columns of U_2 within the N_1-dimensional space.

We next plug in as f_1 the activity f_1^* which minimizes the total error and take the average across the images I. Applying again the calculations underlying Eq. S.4 yields

the estimate

$$\begin{aligned} \langle E(U;I)\rangle_I &= \langle E_{U_1}(f_1^*,I)\rangle_I + E_2\,, \\ E_2 &\geq \tfrac{1}{2}\sum_{k=N_2+1}^{N_1} \lambda_k^{(1)}\,, \end{aligned} \tag{S.6}$$

where $\lambda_i^{(1)}$'s represent the $N_1 - N_2$ smallest eigenvalues of the correlation matrix $\left\langle f_1^* f_1^{*T} \right\rangle_I$. For the first level we have that $E_{U_1}(f_1^*, I) \geq E_{U_1}(U_1^+ I, I)$, so the fact that f_1^* can depend on U_2 does not help to make the first term in (S.6) smaller and hence

$$\langle E_{U_1}(f_1^*,I)\rangle_I \geq \tfrac{1}{2}\sum_{k=N_1+1}^{N_0} \lambda_k^{(0)}\,.$$

In contrast to the single-level optimization, however, it is not clear that the first level lower bound is attained when optimizing the objective function summed across both levels, since this might yield a choice of f_1 which extracts a high penalty in E_2. So in the joint optimization there is a trade-off between the prediction quality in the first level on one hand, and obtaining an easily predictable distribution of f_1 for the second level on the other hand. But if nature does not use the full expressive power provided by the N_1 neurons in level 1, it might quite simply choose a smaller value of N_1 and, instead, use additional neurons to interpret the activity in the second level. From this efficiency perspective, the advantage of miming the sum $E_1 + E_2$ over minimizing E_1 and E_2 individually remains therefore unclear. In contrast, the individual minimization has a distinct advantage that is particularly useful in the sensory representation and that goes beyond the processing speed argument: when information at the different resolution levels needs to be accessed, it is individually optimized at the level of interest. The joint minimization instead may obscure the representation at that level, for the sake optimizing it at the next level.

Reduction to a single level

Given the unclear motivation to consider top-down interactions in terms of image encoding we ask whether we cannot compensate for the top-down signaling by enlarging the interactions within the lower level. This is in fact possible, as alluded to in (S.5). To make this point clear, we express f_1 in new coordinates $\tilde{f}$ (specified below) such that the second term in (S.5) reduces to a sum over squared activities. The 2-layer optimization problem (Eq. S.2 with $L = 2$) then reduces via (S.5) to

$$E(\tilde{U},\tilde{f};I) = \tfrac{1}{2}\|I - \tilde{U}_1\tilde{f}_1\|^2 + \tfrac{1}{2}\sum_{k=N_2+1}^{N_1} (\tilde{f}_1)_k^2\,, \tag{S.7}$$

where N_1 and N_2 are the number of neurons in the first and second level, respectively.

Recognition again amounts to the minimization of (S.7) with respect to the activities $\tilde{f}_1$ of the first-level neurons, while learning amounts to the minimization of (S.7) with respect to $\tilde{U}_1$ at the minimum values of $\tilde{f}_1$. The additional constraint imposed by the joint 2-level minimization is on the mapping $\tilde{U}_1$ that emerges from the specific coordinate transform (see next paragraph). Basically, (S.7) states that the 2-level error function $E_1 + E_2$ is minimized by finding a representation f_1 at the first level that minimizes E_1 while keeping small the $(N_1 - N_2)$ components that are not represented at the second level. Of course, when constructing the first level representation it is not clear which dimensions will be cut off in the second level representations. But instead of

evoking top-down connections from higher levels that would deliver this information – and would also slow down the network relaxation time – we explored an effectively feedforward strategy.

One strategy to mimic the top-down effect in (S.7) is to uniformly keep the f_1 activities small for all components and defer the compression of the f_1-code to the next level. We therefore suggest to directly minimize the quadratic error function E_i at each level i, with an additional regularity constraint of the form $\sum_k (\tilde{f}_i)_k^2$, where the sum now extends across all N_i components of $\tilde{f}_i$, i.e.

$$E_i = \tfrac{1}{2}\|I - U_i f_i\|^2 + \tfrac{\epsilon}{2}\sum_{k=1}^{N_i}(f_i)_k^2\,, \tag{S.8}$$

although with an ϵ smaller than 1/2. This regularity limits the size of the f_i activity, but it does not impose sparseness, as e.g. the L1-norm on f_i would do [3]. We did not consider specific sparseness constraints by several reasons. First, the experimental notion of sparseness is somewhat vague and in biology firing rates are often small but rarely zero, as it would be the case for sparse coding. The square norm in (S.8) impedes high firing rates of individual neurons but allows for uniformly low firing rates across the network. Second, sparseness would lead to strict Gabor-like RFs [3] that, for non-grating stimuli, do not seem to square with the context-dependent RF variations [4–7]. Third, from an economical point of view, it is more efficient to achieve a high stimulus compression rate by limiting the number of f_i neurons, rather by enforcing them mostly not to respond. Finally, abstaining from additional penalty terms increases the clarity of the procedure.

The coordinate transform leading to the form (S.7) is given by $f_1 = S\tilde{f}_1$, where the columns of the orthogonal matrix S are the normalized eigenvectors of the correlation matrix $\left\langle f_1^* f_1^{*T} \right\rangle_I$, ordered according to decreasing eigenvalues (assumed to be non-vanishing). Notice that S can be written as a concatenation $S = U_2 \oplus U_2^{\perp}$ of a $N_1 \times N_2$ dimensional matrix U_2 and a $N_1 \times (N_1 - N_2)$ dimensional matrix $U_2^{\perp}$. Note that the projection matrix $P_{U_2^{\perp}}$ arising in (S.5) transforms into the diagonal matrix $S^T P_{U_2^{\perp}} S$ with 0's in the first N_2 entries and 1's in the $N_1 - N_2$ remaining entries. With $\tilde{U}_1 = U_1 S$ this proves (S.7). The effective receptive fields of the f_1 neurons are given by the non-vanishing row entries of $\tilde{U}_1$, and via the correlation matrix S they depend on the overlaps of the feedforward receptive fields of f_1 and, in the case of the fully-recurrent network, they also depend on the top-down input from the higher level.

S.IV Implementation of modular and cross-level hierarchical coding

The above analysis suggests decoupling the optimization problem for multiple levels, thus enhancing the clarity of the procedure as well as increasing the speed of convergence. Hence, instead of minimizing the overall error across levels (Eq. S.2), we individually minimize the errors (S.1) within each level. For the (negative) gradients with respect to f_i and U_i with $\phi = \mathrm{id}$ we obtain for the modular case,

$$\begin{aligned} \tau\,\dot{f}_i &= -\epsilon f_i + V_i n_i \\ \tau\,\dot{n}_i &= -n_i + f_{i-1} - U_i f_i \\ \Delta U_i &= \eta\, n_i^* \,(f_i^*)^T \end{aligned} \tag{S.9}$$

with $V_i = U_i^T$ and $\Delta V_i = \Delta U_i^T$ (cf. Eqs. (4) and (6) in the main text).

When considering the original, non-modular optimization principle where minimization is performed across all levels as in [2], we need to implement the gradient (S.3). This leads to the same three equations as in (S.9), except the first equation is replaced by

$$\tau\, \dot{f}_i = -\epsilon f_i + V_i n_i - n_{i+1}\,, \tag{S.10}$$

and hence during the learning process f_i's are implicitly modified until the novelty n_{i+1} at the higher level cannot be explained anymore by combining the novelty n_i at level i via matrix V_i (and assuming that ϵ is small, cf. also Fig. Aa). At a functional level this corresponds to the constrained optimization problem for f_i as expressed in Eqs. S.5 and S.7, without the need to consider the activities from the next level $i+1$. An equivalent implementation of the cross-level optimization problem following Eq. S.7 leads to the same dynamics as in (S.9), but with the first equation replaced by a neuronal dynamics with weak and strong neuronal leak terms,

$$\tau\,(\dot{f}_i)_k = \begin{cases} -\epsilon (f_i)_k + (V_i n_i)_k & \text{for } k = 1, \ldots, N_{i+1} \\ -(1+\epsilon)(f_i)_k + (V_i n_i)_k & \text{for } k = N_{i+1}+1, \ldots, N_i\,, \end{cases} \tag{S.11}$$

where N_i and N_{i+1} are the numbers of familiarity neurons at level i and $i+1$, respectively. Thus, recognition is again performed in an effectively feedforward network, with simple (i.e. diagonalized Gaussian) activity priors depending on the number of neurons in the $i+1$'th level (Fig. Ab).

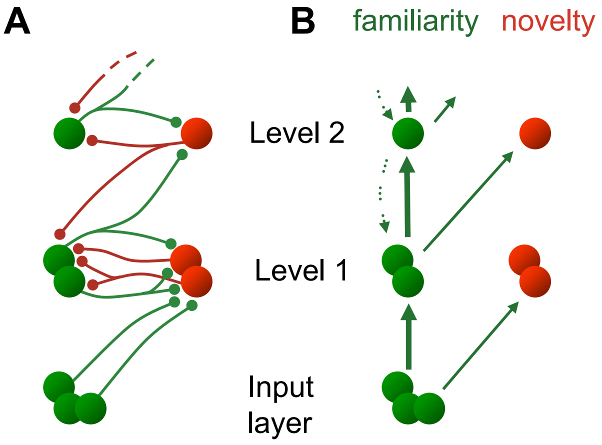

Figure A. Anatomical versus effective connectivity for the cross-level predictive coding as in [2]. **A** In contrast to minimizing the error function locally at each level, minimizing the summed error function across all levels requires top-down connections from the novelty neurons n_{i+1} to the familiarity neurons f_i (cf. Eqs. (S.9), (S.10) and Fig. (2) in the main text). **B** The effective connectivity expressing causal relationships remains feedforward. The only 'top-down' information (dotted arrows) consists in providing the number of familiarity neurons used at level $i+1$, and this determines the number of familiarity neurons at level i which are subject to a strong leak in the neuronal dynamics (cf. Eqs. (S.7) and (S.11)). This distortion caused by the strong leak may reduce the prediction error E_{i+1} at the upper level, while possibly increasing the error E_i at the lower level (with an overall reduction in the sum $E_i + E_{i+1}$).

References

1. Friston K. The free-energy principle: a unified brain theory? Nature Rev Neurosci. 2010;11:127–138.

2. Rao R, Ballard D. Predictive coding in the visual cortex: a functional interpretation of some extra-classical receptive-field effects. Nature Neurosci. 1999;2:79–87.

3. Olshausen BA, Field DJ. Sparse coding with an overcomplete basis set: a strategy employed by V1? Vision Res. 1997;37(23):3311–3325.

4. Carandini M, Demb JB, Mante V, Tolhurst DJ, Dan Y, Olshausen BA, et al. Do we know what the early visual system does? J Neurosci. 2005;25(46):10577–10597.

5. Yeh CI, Xing D, Williams PE, Shapley RM. Stimulus ensemble and cortical layer determine V1 spatial receptive fields. PNAS. 2009;106(34):14652–14657.

6. Victor JD, Mechler F, Ohiorhenuan I, Schmid AM, Purpura KP. Laminar and orientation-dependent characteristics of spatial nonlinearities: implications for the computational architecture of visual cortex. J Neurophysiol. 2009;102:3414–3432.

7. Fournier J, Monier C, Pananceau M, Fregnac Y. Adaptation of the simple or complex nature of V1 receptive fields to visual statistics. Nat Neurosci. 2011;14(8):1053–1060.
